# Ligature of Common Carotid Artery in an Abscess Following Diphtheritic Scarlatina; Recovery

**Published:** 1887-06

**Authors:** 


### Cullings from Contemporaries.

### A CASE OF LIGATURE OF THE COMMON CAROTID IN AN ABSCESS FOLLOWING DIPHTHERITIC SCARLATINA, RESULTING IN RECOVERY.

*Translation from Centralblatt—Kinderheilkunde.*

A girl, nine years of age, with hereditary taint, was taken sick with scarlet fever, diphteria, and swelling of the right lymphatic glands. Suppuration set in, in the latter, and the abscess was relieved by means of a small incision. After some days the wound began to bleed profusely, ceasing only upon compression of the carotid. The next morning S. saw the child for the first time, and proceeded on the same day to ligate the carotid. After splitting the abscess and removing the coagula, S. found in the region of the great horn of the hyoid, a small cavity out of which slowly trickled light colored blood. It must doubtlessly therefore have been the carotid itself, or one of its terminal branches near its point of exit must have been eroded. [sloughed?]. The child having already suffered much loss of blood was too weak to risk the delay of ligature in loco, therefore the carotid communis was ligated in the elevation of the cartilage ring, the wound then well cleansed and tamponed with iodoformed cotton. Five days after the operation the child had sudden attacks of delirium and convulsions of the left half of the face and body, to which was added shortly after, a total paresis of the left side, which in its turn was followed by a complete paralysis of both left extremities. Soon after there was an apparent motion in the paralysed leg, and in two weeks the limb had almost entirely regained its function of mobility. The left hand recovered much more slowly, and then only partially, and a half year after the ligation of the carotid, there remained a paresis of the extensor muscles of the left hand and the peronial muscles of the left foot; also the appearances of an imperfect nourishment of the cortex of the brain, the child appearing less mentally developed after its illness than other children of a similar age. Previous to the illness the child's mind was perfectly developed. The author thinks that perhaps both the carotid ligation and the diphtheria were the causes of the brain disturbance.

*Lunin, Petersburg.*

